# Factors affecting human colostrum fatty acid profile: A case study

**DOI:** 10.1371/journal.pone.0175817

**Published:** 2017-04-14

**Authors:** Vassilia J. Sinanoglou, Dionisis Cavouras, Theodora Boutsikou, Despina D. Briana, Dimitra Z. Lantzouraki, Stella Paliatsiou, Paraskevi Volaki, Sotiris Bratakos, Ariadne Malamitsi-Puchner, Panagiotis Zoumpoulakis

**Affiliations:** 1 Laboratory of Chemistry, Analysis & Design of Food Processes, Instrumental Food Analysis, Department of Food Technology, Technological Educational Institution of Athens, Greece; 2 Medical Image and Signal Processing Laboratory, Department of Biomedical Engineering, Technological Education Institution of Athens, Greece; 3 Department of Neonatology, National and Kapodistrian University of Athens, Medical School Aretaieio University Hospital, Athens, Greece; 4 Institute of Biology, Medicinal Chemistry and Biotechnology, National Hellenic Research Foundation, Athens, Greece; University of Illinois, UNITED STATES

## Abstract

The role of maternal colostrum to infant development has been extensively studied and presented. Among the main factors which contribute to breast milk composition are maternal diet, age and body mass index, parity, duration of pregnancy and stage of lactation. This study aims to investigate the potential impact of several factors including demographic (i.e. maternal age and nationality) on the colostrum fatty acid profile. Colostrum was collected the third day postpartum in a Greek maternity hospital. Certain lipid quality indices and fatty acid ratios were estimated and results were statistically processed. The main identified fatty acids were palmitic (C16:0), oleic (C18:1ω-9), and linoleic (C18:2ω-6) acids. Among fatty acids, saturated fatty acids predominated (47.61%), followed by monounsaturated fatty acids (39.26%), while polyunsaturated fatty acids had the lowest proportion (13.13%). Values of lipid quality indices were within the reported in the literature ranges. Maternal body mass index, nationality, age, mode of delivery, gender and fetal weight percentile were studied in respect to their potential influence on the fatty acid profile of colostrum fat. Results suggest that colostrum fatty acid profile was mainly dependent on maternal nationality and age rather than mode of delivery and maternal BMI. Regarding the effect of maternal nationality, significant differences were found for saturated and monounsaturated fatty acids. Of the most interesting findings is that colostrum fat from older (≥35 years) mothers had less saturated fat and more appropriate LQIs values. Finally, a reversed correlation was observed between the customized centile of the infants and the colostrum fat content.

## Introduction

Maternal milk contains all the nutrients an infant needs in the right amounts and in an easily absorbable form. More specifically, it contains more than 200 functional components, including lipids, various proteins, enzymes, carbohydrates, vitamins, minerals and macronutrient elements.[1] Furthermore, it contains growth factors and hormones to assist development.[1] Several studies have reported that maternal milk has anti-infective and anti-inflammatory properties and its composition changes as the baby grows.[1,2]

Maternal colostrum and mature milk contain 1.9–2.3% and 3.5–4.5% of lipids respectively, comprising mostly triglycerides.[3] The most abundant saturated (SFA) and monounsaturated (MUFA) fatty acids in maternal milk triglycerides are palmitic (C16:0) and oleic (C18:1ω-9) acids followed by the essential linoleic (C18:2ω-6) and alpha-linolenic (C18:3ω-3) acids.[3] Arachidonic (C20:4ω-6), eicosatrienoic (C20:3ω-6), docosahexaenoic (C22:6ω-3) (DHA) and eicosapentaenoic (C20:5ω-3) (EPA) acids are also identified in minor proportions.[4] Long chain fatty acids (LC-FA) in maternal milk originate either from maternal diet or originate from adipose tissue and liver metabolism; whereas short and medium chain fatty acids are synthesized *de novo* within the mammary gland.[2,5]

Main factors affecting breast milk lipid composition are maternal age, parity, duration of pregnancy, maternal body mass index (BMI), maternal diet, stage of lactation, daily breastfeeding rate, but also presence or development of diabetes during pregnancy and maternal nationality.[1,4,6–10]

The purpose of this research was to investigate the effect of maternal nationality, age, BMI, gestational age, mode of delivery, infantile gender, customised centile, on the fatty acid profile of colostrum in a cohort of women living and delivering in Greece. Moreover, a further goal was to estimate lipid quality indices (LQI) (e.g. ω-6/ω-3 fatty acids ratios, atherogenic, thrombogenic and peroxidisability indices) which reflect the functionality of the maternal milk.

## Materials and methods

### Chemicals, standards, and solvents

The standards for determining fatty acid methyl esters were: Supelco TM 37 Component FAME Mix C4-C24, Supelco PUFA No.1, Marine Source and conjugated linoleic acid methyl esters standard mixture (Sigma-Aldrich Company, St. Louis, MO, USA). All solvents used for GC-FID analyses were of HPLC grade from Merck (Darmstadt, Germany). All reagents used were of analytical grade and they were purchased from Mallinckrodt Chemical Works (St. Louis, MO, USA) and from Sigma Chemical Co (Sigma-Aldrich Company, UK).

### Colostrum sampling

Following informed signed consent, this study comprised 120 women aged 24 to 42 years, being at the third postpartum day. The study was approved by the Ethical Committee of our teaching hospital, Aretaieio University Hospital. By ending breastfeeding, between 12:00 and 13:00 hours, participating women, expressed with a manual pump from one breast, colostrum. A 3 mL aliquot was collected in a sterile glass vial and stored at -25°C for further analysis. A total of 97 samples were finally collected for the study. Demographic data applying to mothers and infants (e.g. maternal nationality, age, parity, BMI, gestational age, mode of delivery, infantile gender, birth weight, customised centile) were collected by filling a relative questionnaire. The study was approved by the Ethical Committee of our teaching hospital. Table 1 summarizes the groups and number of samples for each studied factor as depicted from the completed questionnaires.

**Table 1:** Categorization of colostrum samples from lactating women according to studied factors and groups (n = 97).

	Groups			Number of samples
Factors	1	2	3	Ν1-Ν2-Ν3
Maternal nationality	Greek	Albanian	Other	51–22–24
Maternal age (years)	< 30	30–34	≥ 35	25–41–31
Mode of delivery	Vaginal	Cesarean		41–56
Gestational age (weeks)	38.74±0.80	38.95±0.99	36.73±0.59	41–41–15
Maternal Body mass index (BMI) (kg/m^2^)	< 26 normal	26–30 overweight	> 30 obese	35–37–25
Customized centile	< 20	20–70	>70	24–45–28
Gender	Male (N1 = 59)		Female (N2 = 38)	

### Total lipid extraction

Total lipids of homogenized raw milk samples were extracted according to a modification of the Folch method.[11] In detail, in a tube 10 mL of chloroform-methanol solution (2:1 by vol.) was added to 1.2 mL of milk. The mixture was homogenized in a vortex for 5 min and then centrifuged for 5 min (3,000 rpm) at 5°C. The upper layer was obtained and the procedure was repeated. The combined supernatants were transferred to a separating funnel and an appropriate volume of water was added to a final ratio of 2:1:0.6 (by vol.) in chloroform-methanol-water. The new mixture was homogenized in a vortex for 5 min and allowed to stand until phase separation, and the lower lipid layer was obtained. The lipid layer was evaporated to dryness in a rotary evaporator to constant weight, the lipid content was determined gravimetrically, then re-dissolved in chloroform/methanol (9:1, v/v) and finally stored at -10 ^o^C until further use. To prevent oxidation, t-butyl-hydroquinone was added to all samples during preparation.

### Gas chromatography analysis of fatty acid methyl esters

The analysis of fatty acid methyl esters (FAME) was performed according to Sinanoglou *et al*.,[12] using an Agilent 6890 Series Gas Chromatograph (Agilent Technologies, Palo Alto, CA) equipped with flame ionization detector. The identification and the relative content of fatty acids in each samplewere determined according to the procedure described by Sinanoglou *et al*.[12]

### Calculations of Indices

The atherogenic index (AI) and thrombogenic index (TI) were calculated according to the Ulbricht and Southgate[13] equations:

$$AI=\frac{\left[12:0+(4*14:0)+16:0\right]}{\omega 3PUFA+\omega 6PUFA+MUFA}$$ (1)

$$TI=\frac{\left[14:0+16:0+18:0\right]}{0.5*MUFA+0.5*\omega 6PUFA+3*\omega 3PUFA+\frac{\omega 3PUFA}{\omega 6\;PUFA}}$$ (2)

The peroxidisability index (PI) was calculated according to the equation proposed by Erickson:[14]

PI=(0.025*monoenes)+(1*dienes)+(2*trienes)+(4*tetraenes)+(6*pentaenes)+(8*hexaenes) (3)

Hypocholesterolaemic (hI) and hypercholesterolaemic (HI) fatty acids were calculated according to the Santos-Silva *et al*.[15] equations:

hI=C18:1ω9+C18:2ω6+C20:4ω6+C18:3ω3+C20:5ω3+C22:5ω3+C22:6ω3 (4)

HI=C14:0+C16:0 (5)

### Statistical analysis

Measurements were obtained in triplicate and averaged values of fatty acids were calculated. Statistical analysis consisted of employing the Kruskall-Wallis non-parametric test for determining significant differences of the parameters (FA, LQI, FA ratios) in the following demographic factors (maternal nationality, BMI and age, mode of delivery, customized centile) and the Wilcoxon non-parametric test in the two class problem (infantile gender). A P value < 0.05 was considered to be statistically significant. Non-parametric tests were employed since normality in data distributions could not be proved. In the 3-class problems, when statistical significant differences were revealed amongst classes, a further step was undertaken, that of determining which pairs of classes sustained statistical significant differences. The statistical tests were conducted employing Matlab functions, which are well established procedures of statistical analysis. Analysis was performed using Matlab software (Matlab version 6.04, The MathWorks, Inc., Natick, Massachusetts, United States).

## Results and discussion

### Fatty acids profile and lipid quality indices values of colostrum

Gas chromatography (GC-FID) analysis of the colostrum total lipid revealed the presence of 49 fatty acids (FA). The health impact and functionality of the colostrum was evaluated by: a) the main fatty acids identified, such as palmitic, stearic, oleic and linoleic acids, b) the calculation of fatty acids ratios, such as ω-6/ω-3, monounsaturated/saturated (MUFA/SFA) and polyunsaturated/saturated (PUFA/SFA) and c) the lipid quality indices (LQI) assessment, such as atherogenic (AI), thrombogenic (TI), peroxidisability (PI), hypercholesterolaemic (HI) and hypocholesterolaemic (hI) indices, all presented in Table 2.

**Table 2:** Means of total lipids [% (w/w) wet weight], fatty acids’ composition [% (w/w) of TFA] in total lipids and lipid quality indices’ values (±S.E.M.) of colostrum (n = 97).

Fatty acid	Mean	S.E.M.	Fatty acid	Mean	S.E.M.	Fatty acid	Mean	S.E.M.
Total lipid	1.90	0.09	C18:1ω-7	2.13	0.21	C22:4ω-6	0.17	0.02
C8:0	0.16	0.05	C182*c*9*t*11	0.13	0.02	C22:5ω-6	0.10	0.02
C10:0	1.05	0.08	C182*t*9*t*11	0.05	0.01	C22:5ω-3	0.18	0.04
C10:1	0.10	0.02	C182*t*10*c*12	0.08	0.01	C24:0	0.08	0.01
C12:0	5.06	0.12	C182*t*11*c*13	0.06	0.01	C22:6ω-3	0.15	0.01
C13:0	0.04	0.01	C182*t*11*t*13	0.07	0.01	C24:1ω-9	0.08	0.01
C14:0	6.85	0.18	C18:2ω-6	9.94	0.18	SFA	47.61	0.48
C14:1	0.15	0.01	C18:3ω-6	0.12	0.02	MUFA	39.26	0.42
*iso*C15:0	0.02	0.00	C18:3ω-3	0.33	0.02	PUFA	13.13	0.21
C15:0	0.36	0.01	C18:4ω-3	0.12	0.01	ω-6	11.38	0.19
C15:1ω-5	0.07	0.01	C20:0	0.13	0.01	ω-3	1.36	0.05
C16:0	26.71	0.24	C20:1ω-9	0.59	0.02	MUFA/SFA	0.82	0.02
*iso*C16:0	0.36	0.03	C20:2ω-6	0.05	0.00	PUFA/SFA	0.24	0.01
C16:1ω-7	1.30	0.08	C20:3ω-6	0.52	0.01	MUFA/PUFA	2.99	0.06
*iso*C17:0	1.16	0.11	C20:4ω-6	0.47	0.02	ω-6/ω-3	8.37	0.38
*anteiso*C17:0	0.09	0.01	C20:3ω-3	0.56	0.02	AI	1.17	0.03
*cyclo*C17:0	0.07	0.00	C21:0	0.02	0.02	TI	1.33	0.02
C17:0	0.40	0.09	C20:5ω-3	0.02	0.00	hI	45.73	0.49
C17:1	0.13	0.01	C22:0	0.07	0.00	HI	33.58	0.32
C18:0	4.95	0.09	C22:1ω-9	0.06	0.00	h/H	1.38	0.02
C18:1*t*11	0.01	0.00	C22:2ω-6	0.01	0.00	PI	20.44	0.47
C18:1ω-9	34.64	0.45	C23:0	0.03	0.01			

Results represent mean values ± S.E.M. (standard error of mean) (n = 97 separate samples)

ΑΙ = atherogenic index; hI = hypocholesterolaemic fatty acids index; HI = hypercholesterolaemic fatty acids index; h/H = hypocholesterolaemic/ hypercholesterolaemic fatty acids; MUFA = monounsaturated fatty acids; PI = peroxidisability index; PUFA = polyunsaturated fatty acids; SFA = saturated fatty acids; TI = thrombogenic index.

Saturated fatty acids (SFA) approximately constituted 47.6% of the total fatty acids. Moreover, monounsaturated fatty acids (MUFA) were second in abundance and were found three times greater than polyunsaturated fatty acids (PUFA) (Table 2). The SFA proportion of the examined colostrum samples was higher than that reported by Silva *et al*.[16] for the mature breast milk of Brazilian women (39.7%), by Fares *et al*.[17] for the preterm colostrum of Tunisian women (39.6%), and by Nishimura *et al*.[18] for the mature breast milk of women living far from the coastal area of Brazil (42.67%). Nevertheless, it was similar to that reported by Antonakou *et al*.[19] for Greek maternal milk during the first 6 months of exclusive breast feeding (45.67%) and by Kuipers *et al*.[20] for colostrum in a sub-Saharan population with high fish consumption (45.8%), but lower to that reported by Kuipers *et al*.[20] for transitional and mature milks in a sub-Saharan population with high fish consumption (58.1 and 59.2%, respectively).

Palmitic acid was the main SFA, representing more than 26% of total fatty acids, followed by myristic and stearic acids (Table 2). The odd- branched- and *cyclo*-chain SFA, such as C15:0, *iso*C15:0, *iso*C16:0, C17:0, *iso*C17:0, *anteiso*C17:0 and *cyclo*C17:0, were also identified in minor proportions. This finding is of value for the infant, as C15:0 and C17:0 have been shown to have an important inverse association with the incidence of coronary heart disease and type II diabetes[21] later in life.

Regarding MUFA, oleic acid contributed the most to their total proportion. It is important to highlight the identification of the C18:1*t*11 (vaccenic acid, VA) as well as of the conjugated linoleic acids (CLA) isomers, C18:2*c*9*t*11 (rumenic acid), C18:2*t*9*t*11, C18:2*t*10*c*12, C18:2*t*11*c*13 and C18:2*t*11*t*13 in minor proportions, all constituting almost 0.4% of the total fatty acids (Table 2). According to Larqué *et al*.,[22] *trans* fatty-acid content in maternal milk presents high variability, ranging from 7.2% in Canada to 0.95% in Spain. The presence of *trans* fatty acids in maternal milk, results from the consumption of partially hydrogenated fats, processed and fried foods as well as dairy products. In the present study, it becomes obvious that the very low proportion of *trans* fatty-acid in colostrum fat is related to very low dietary intakes of partially hydrogenated oils, reducing significantly the exposure of the infant to these fatty acids.

Linoleic acid was the most dominant among PUFA. Alpha-linolenic (C18:3ω-3), arachidonic (C20:4ω-6), eicosatrienoic (C20:3ω-3) and dihomo-γ-linolenic (C20:3ω-6) acids were present in modest amounts (> 0.30%), whereas gamma-linolenic (C18:3ω-6), stearidonic (C18:4ω-3), adrenic (C22:4ω-6), docosapentaenoic (C22:5ω-3, DPA) and docosahexaenoic (C22:6ω-3, DHA) acids were also found in negligible amounts (< 0.20%) (Table 2).

The ω-6/ω-3 ratio, which indicates the balance between the essential fatty acids, was 8.37. Interestingly, LC-PUFA of ω-6 and ω-3 families accounted for 1.31 and 0.91% of the total fatty acids respectively (data calculated from Table 2), whereas their ratio (1.44) was much lower than the European range (2.23–3.19%).[23]

Although the lipid and fatty acid requirements for newborns may differ from those of the adults it is of interest to state that maternal colostrum fat had relatively low atherogenic and thrombogenic indices (1.17 and 1.33 respectively), which is lower than the corresponding indices for cow milk and similar to previous studies.[24,25] Furthermore, the relatively high hypocholesterolaemic/hypercholesterolaemic (h/H) ratio (1.38) is desirable, because the higher the values of h/H ratio, the lower the proportion of hypercholesterolaemic fatty acids C14:0 and C16:0 and their effect on low-density lipoprotein (LDL) increase.[25]

### Effect of maternal age

Increased maternal age at delivery is nowadays common in Greece and generally in western countries; therefore it is interesting to mention the association of maternal age with breast milk fatty acid profile. The effect of maternal age on the individual FA proportions, sums and ratios, as well as the LQI values of colostrum fat is depicted in Table 3. Colostrum fat from mothers above 35 years old, had significantly lower SFA and higher MUFA proportions compared to colostrum of younger mothers (*P<*0.05). Furthermore, the ω-6/ω-3 ratio, as well as the atherogenic and thrombogenic indices in colostrum fat showed a significant decrease, whereas h/H ratio a significant increase in parallel with maternal age (*P<*0.05) (Table 3). Specifically, proportions of lauric (C12:0) and myristic (C14:0) acids were highest in younger (<30 years) mothers (*P* = 0.006 for lauric and 0.011 for myristic). According to literature reports, high proportions of lauric and myristic acids in maternal milk fat could be attributed to a high-carbohydrate diet.[26] In addition, lauric and myristic acids are shown to have total cholesterol raising effect when compared to other saturated fatty acids.[27] Nevertheless, lauric and myristic acids, which can be synthesized *de novo* within the mammary gland, provide energy, as well as viral and microbial protection to infants.[28] Mothers aged 30–34 years presented the highest proportions for palmitic (C16:0), stearic (C18:0) and arachidic (C20:0) acids (*P* = 0.001, 0.000 and 0.001, respectively). The proportions of palmitic and stearic acids, in maternal milk fat, are significant indicators for red meat, poultry and dairy products consumption by mothers.[29] Furthermore, *iso*C16:0 and C16:1ω-7 proportions were significantly increased (*P* = 0.001 and 0.000, respectively) in older (≥35 years) mothers. The same applied for heptadecanoic acid (C17:0) proportion, which was also higher (*P* = 0.031) in older (≥35 years) mothers. This finding is interesting, as convincing evidence exists that C17:0 might reverse the early stages of diabetes in humans.[21] Conjugated linoleic acids (CLA) isomers’ C18:2*t*10*c*12, C18:2*t*11*c*13 and C18:2*t*11*t*13 proportions (*P* = 0.045, 0.005 and 0.026, respectively) were also higher in mothers aged 30–34 years. CLA have been shown to exert beneficial physiological functions against chronic diseases such as cancer or indices of the metabolic syndrome as diabetes, hypertension and obesity.[30–32] In this respect, the higher breast milk proportions of CLA in this population could possibly reflect a counterbalance mechanism against cardiovascular risk for the mother-infant dyad.

**Table 3:** Effect of maternal age on the individual FA proportions, sums and ratios, as well as the LQI values of colostrum fat.

Groups		1	2	3			
	*P<*0.050	< 30	30–34	≥ 35	'1–2'	'1–3'	'2–3'
C12:0	0.01060	5.64±0.21	4.74±0.11	5.02±0.16	*	*	
C14:0	0.00619	7.36±0.02	6.82±0.21	6.48±0.15	*	*	
C16:0	0.00142	26.80±0.30	27.35±0.20	25.84±0.22			*
*iso*C16:0	0.00068	0.35±0.03	0.26±0.03	0.50±0.04		*	*
C16:1ω-7	0.00015	1.25±0.07	1.06±0.07	1.64±0.10			*
C17:0	0.03126	0.26±0.03	0.21±0.01	0.76±0.14			*
C18:0	0.00000	4.81±0.08	5.28±0.07	4.61±0.10	*		*
C18:1ω-9	0.01478	33.10±0.54	34.99±0.31	35.44±0.50	*	*	
C18:2*t*10*c*12	0.04501	0.06±0.00	0.10±0.01	0.07±0.01	*		
C18:2*t*11*c*13	0.00472	0.06±0.00	0.06±0.00	0.06±0.00		*	*
C18:2*t*11*t*13	0.01563	0.06±0.00	0.09±0.01	0.06±0.00	*		*
C20:0	0.00111	0.12±0.00	0.14±0.00	0.12±0.00	*		*
SFA	0.03268	48.36±0.44	47.72±0.45	46.66±0.54		*	
MUFA	0.01620	38.39±0.41	39.06±0.34	40.12±0.51		*	*
MUFA/SFA	0.03609	0.81±0.02	0.83±0.01	0.88±0.02		*	
ω-6/ω-3	0.02494	9.48±0.26	9.06±0.21	8.93±0.58		*	*
AI	0.01125	1.23±0.03	1.18±0.03	1.10±0.03		*	
TI	0.00142	1.37±0.03	1.37±0.02	1.25±0.02		*	*
HI	0.00168	34.16±0.38	34.17±0.30	32.32±0.26		*	*
h/H	0.02159	1.33±0.03	1.37±0.02	1.45±0.02		*	*

Results represent mean values ± S.E.M. (standard error of mean)

Mean values in the same row with * differ significantly between two specific groups of maternal age (< 30, 30–34, ≥ 35) with *P<*0.05

Taken all above results, some important conclusions can be drawn. Colostrum fat from older (≥35 years) mothers had less saturated fat and more appropriate values of LQIs. Significant differences in heptadecanoic acid proportion of human milk fat, is probably dependent on the consumption of dairy fats.[33] Furthermore, the presence of higher proportions of SFA and *trans* fatty acids in colostrum of younger (<35 years) mothers, is possibly associated with the different dietary habits of younger women and the more frequent consumption of ready and processed meals. The presence of lauric acid in younger women colostrum fat, which as discussed has atherogenic effect in human diet, could be compensated by its multiple roles as an antibacterial and antiviral agent.[34]

### Effect of body mass index (BMI)

The effect of maternal BMI on the individual FA proportions, sums and ratios, as well as the LQI values of colostrum fat is presented in Table 4. Maternal BMI is positively related to infant obesity risk.[35] In addition, obesity presents with impaired regulatory metabolic adaptations in various stimuli, including fasting, exercise and breastfeeding.[36–38] In study animals, prenatal obesity has been linked to impaired development of the mammary gland, lactation and offspring growth.[39,40] In our study, colostrum fat content in overweight mothers was found significantly (*P<*0.05) lower as compared to the fat content of normal weight and obese mothers. Moreover, the proportions of decanoic (C10:1), tridecanoic (C13:0) and adrenic (C22:4ω-6) acids were higher (*P* = 0.000, 0.002 and 0.016, respectively) in obese mothers. Interestingly, obese and overweight mothers had significantly higher (*P<*0.05) ω-3 PUFA in colostrum fat as compared to normal weight mothers. In addition, docosadienoic acid (C22:2ω-6) was highest in the colostrum fat of normal weight mothers, as compared to the two other groups (*P* = 0.000). Also the alpha-linolenic acid (C18:3ω-3) proportion was significantly (*P* = 0.001) higher in the colostrum fat of overweight mothers as compared to the other two groups. Proportions of conjugated linoleic acids isomers C18:2*c*9*t*11 (rumenic acid) and C18:2*t*11*t*13 were higher (*P* = 0.028 and 0.017, respectively) in the colostrum fat of normal weight mothers.

**Table 4:** Effect of maternal BMI on the individual FA proportions, sums and ratios, as well as the LQI values of colostrum fat.

Groups		1	2	3			
	*P<*0.050	< 26 normal	26–30 overweight	> 30 obese	'1–2'	'1–3'	'2–3'
Fat	0.00240	2.03±0.08	1.71±0.10	2.01±0.09	*		*
C10:1	0.00008	0.04±0.01	0.09±0.01	0.19±0.04	*	*	
C13:0	0.00180	0.01±0.00	0.04±0.01	0.07±0.01	*	*	
C18:2*c*9*t*11	0.01851	0.19±0.04	0.07±0.01	0.13±0.02	*	*	
C18:2*t*11*t*13	0.00718	0.09±0.01	0.06±0.01	0.07±0.01	*		
C18:3ω-3	0.00053	0.30±0.01	0.34±0.01	0.30±0.01	*	*	*
C22:2ω-6	0.00007	0.02±0.00	0.00±0.00	0.00±0.00	*	*	
C22:4ω-6	0.00601	0.15±0.01	0.14±0.01	0.27±0.03		*	*
ω-3	0.02581	1.23±0.03	1.38±0.05	1.48±0.08		*	

Results represent mean values ± S.E.M. (standard error of mean)

Mean values in the same row with * differ significantly between two specific groups of maternal BMI (normal, overweight, obese) with *P<*0.05

The aforementioned results pinpoint distinct differences in the fatty acid composition of breast milk between obese/overweight and normal weight mothers. In our study mothers with increased BMI presented with higher proportions of ω-3 PUFA (as alpha-linolenic acid) and adrenic acid in colostrum fat. The impact of BMI on human gut microbiota modulation is long established.[41] MUFA/PUFA rich diets are known to affect the gut microbiota,[42] while the ω-6/ω-3 ratio in human milk has increased 3-fold over the last 30 years, reflecting the dietary changes along with the obesity epidemic and resulting in accumulation of adipose tissue in breastfeeding infants of mothers with increased BMI.[43] On the other hand PUFA have been linked to a favourable metabolic profile, resulting to reduction of cardiovascular risk.[44] In this respect, their increased proportions in the milk of overweight or obese mothers could possibly indicate a cardioprotective mechanism for their offspring.

### Effect of nationality of the mother

Considering the different dietary patterns of women of different nationalities, it is interesting to compare colostrum fatty acid profile and estimate possible influencing factors. The effect of maternal nationality on the individual FA proportions, sums and ratios as well as the LQI values of colostrum fat is presented in Table 5. Total saturated fatty acids (SFA), atherogenic and thrombogenic indices and ω-6/ω-3 ratio in colostrum fat were significantly (*P* = 0.001, 0.000, 0.001 and 0.016, respectively) lower in Greek mothers as compared to the corresponding fat of mothers of different nationalities. Arguably, this health related finding is of great interest, as the lower ω-6/ω-3 ratio is an important determinant of reduced risk for many diseases. Additionally, a healthy diet is associated with low atherogenic and thrombogenic indices. Furthermore, colostrum fat from Greek mothers had significantly higher oleic (C18:1ω-9) acid, eicosanoic (C20:1ω-9) acid and MUFA proportions as well as hypocholesterolaemic/hypercholesterolaemic (h/H) ratio (*P<*0.001) as compared to the corresponding fat of Albanians and mothers of other nationalities. This result is of value because MUFA and especially oleic acid constitute the predominant energy source for the suckling infant. It is of importance to point out that the mean proportion of SFA and MUFA of colostrum fat from Greek women (46.27 and 40.33% of total fatty acids) in contrast to the one of mothers of other nationalities, fell within the European range (37.24–46.88% and 39.11–45.19%, respectively).[23] This is probably related to the Mediterranean diet, which contains a low proportion of SFA due to the high intake of vegetables, grains and legumes. Comparing the colostrum fat differences among Greeks, Albanians and mothers of other nationalities, in oleic and linoleic acids’ proportions (Table 5), it seems that Greeks consumed higher portions of olive oil whereas the diets of mothers of other nationalities contained higher portions of vegetable oils such as sunflower seed, soybean, corn or other seed oils, which are high in linoleic acid. Moreover, the colostrum fat of Albanians contained the highest proportions (*P<*0.05) of lauric (C12:0) and myristic (C14:0) acids. According to Nasser *et al*.[29] the high consumption of carbohydrates is likely to increase C12:0 and C14:0 proportions in human milk, via the conversion of carbohydrates into medium-chain SFA in the human breast.

**Table 5:** Effect of maternal nationality on the individual FA proportions, sums and ratios as well as the LQI values of colostrum fat.

Groups		1	2	3			
	*P<*0.050	Greek	Albanian	Other	'1–2'	'1–3'	'2–3'
C12:0	0.04153	4.69±0.11	5.56±0.15	5.39±0.12	*		
C14:0	0.00021	6.38±0.16	7.74±0.24	7.04±0.13	*	*	
C14:1	0.00000	0.13±0.01	0.18±0.01	0.17±0.00	*	*	
C15:0	0.02002	0.36±0.02	0.39±0.01	0.34±0.01	*		
C16:0	0.02425	26.23±0.21	27.52±0.30	27.05±0.23	*		
*iso*C17:0	0.01090	0.96±0.10	1.15±0.11	1.58±0.13		*	
*anteiso*C17:0	0.04664	0.08±0.00	0.10±0.01	0.09±0.00	*		
*cyclo*C17:0	0.00593	0.07±0.00	0.09±0.00	0.06±0.00	*		*
C17:0	0.00006	0.54±0.12	0.33±0.03	0.16±0.01		*	*
C18:0	0.01529	5.04±0.09	5.01±0.09	4.68±0.08		*	
C18:1ω-9	0.00001	35.78±0.44	32.50±0.52	34.19±0.31	*	*	
C18:2*t*10*c*12	0.00003	0.10±0.02	0.07±0.00	0.05±0.00		*	*
C18:2*t*11*c*13	0.00059	0.06±0.00	0.10±0.02	0.04±0.00	*	*	*
C18:2ω-6	0.03873	9.93±0.16	9.30±0.18	10.54±0.20			*
C20:1ω-9	0.00004	0.63±0.02	0.55±0.01	0.54±0.01	*	*	
C20:3ω-3	0.01287	0.59±0.02	0.51±0.02	0.54±0.02	*		
SFA	0.00073	46.27±0.43	49.81±0.59	48.17±0.38	*	*	
MUFA	0.00027	40.33±0.41	37.58±0.44	38.39±0.37	*	*	
MUFA/SFA	0.00039	0.89±0.02	0.77±0.02	0.80±0.01	*	*	
ω-6/ω-3	0.00649	8.69±0.36	8.52±0.23	10.59±0.48		*	*
AI	0.00018	1.08±0.02	1.34±0.04	1.19±0.02	*	*	
TI	0.00144	1.26±0.02	1.47±0.04	1.36±0.02	*	*	
hI	0.00010	46.91±0.46	42.90±0.61	45.81±0.32	*		*
HI	0.00015	32.61±0.26	35.26±0.41	34.10±0.27	*	*	
h/H	0.00029	1.45±0.02	1.25±0.03	1.36±0.02	*	*	

Results represent mean values ± S.E.M. (standard error of mean)

Mean values in the same row with * differ significantly between two specific groups of maternal nationality (Greek, Albanian, Other) with *P<*0.05

### Effect of gestational age and mode of delivery

The role of gestational age to the fatty acid profile of colostrum fat was also studied emphasizing in colostrum from preterm and term births. The effect of gestational age and mode of delivery on the individual FA proportions, sums (MUFA, PUFA) and ratios (MUFA/SFA, MUFA/PUFA), as well as the LQI values of colostrum fat, is depicted in Table 6. Palmitic acid (C16:0) proportion and HI value decreased significantly (*P* = 0.001 and 0.031, respectively) from term to preterm colostrum. Furthermore, preterm colostrum fat had significantly higher sum of PUFA, ω-6 PUFA, gamma-linolenic (C18:3ω-6) and adrenic (C22:4ω-6) acids proportions as well as peroxidisability index (PI) value (*P* = 0.002, 0.015, 0.042, 0.018 and 0.000, respectively) compared with the term colostrum fat. This finding is in accordance to previous ones showing higher long-chain PUFA proportion in preterm milk compared to full-term milk.[17,45] Furthermore, the above observation is of value, because preterm infants have limited capacity for elongating and desaturating linoleic and alpha-linolenic acids to long-chain PUFA and negligible stocks of them exist in their adipose tissue.[46] This finding may demonstrate the different response of the organism in view of a risk factor, increasing the quality of colostrum fat to cover specific needs of preterm infants.

**Table 6:** Effect of gestational age and mode of delivery on the individual FA proportions, sums and ratios as well as the LQI values of colostrum fat.

Groups		1	2	3			
	*P<*0.050	38.74±0.80	38.95±0.99	36.73±0.59	'1–2'	'1–3'	'2–3'
C12:0	0.01765	5.28±0.12	4.50±0.08	5.88±0.18	*		*
C14:0	0.00082	7.43±0.20	6.30±0.16	6.89±0.17	*		
C16:0	0.00143	26.89±0.26	27.15±0.20	25.43±0.26		*	*
C18:1ω-9	0.00009	33.44±0.47	36.40±0.29	33.20±0.59	*		*
C18:3ω-6	0.04246	0.12±0.03	0.07±0.01	0.22±0.03			*
C18:3ω-3	0.02833	0.36±0.02	0.29±0.01	0.32±0.01	*		*
C18:4ω-3	0.00323	0.14±0.01	0.11±0.00	0.10±0.00	*	*	
C20:0	0.00127	0.15±0.01	0.12±0.00	0.12±0.01	*	*	
C20:4ω-6	0.00646	0.48±0.01	0.43±0.02	0.50±0.02	*		*
C20:5ω-3	0.04829	0.02±0.00	0.02±0.00	0.03±0.00	*		
C22:4ω-6	0.00866	0.18±0.02	0.12±0.01	0.27±0.04	*		*
MUFA	0.00076	38.22±0.46	40.73±0.29	37.93±0.48	*		*
PUFA	0.00249	13.29±0.23	12.77±0.19	14.15±0.21		*	*
ω-6	0.00506	11.34±0.21	11.02±0.16	12.22±0.18		*	*
ω-3	0.03460	1.42±0.05	1.23±0.03	1.48±0.08	*		
MUFA/SFA	0.01473	0.81±0.02	0.89±0.01	0.81±0.02	*		*
MUFA/PUFA	0.00003	2.95±0.05	3.26±0.05	2.75±0.06	*	*	*
AI	0.03605	1.25±0.04	1.09±0.02	1.17±0.03	*		
hI	0.01338	44.53±0.53	47.20±0.34	44.90±0.63	*		
HI	0.03070	34.32±0.38	33.44±0.26	32.32±0.28		*	*
PI	0.00033	20.69±0.46	18.99±0.31	23.18±0.65	*		*

Results represent mean values ± S.E.M. (standard error of mean)

Mean values in the same row with * differ significantly between two specific groups of gestational age and mode of delivery with *P<*0.05

However, when considering the mode of delivery of term infants, it was found that the proportions of lauric (C12:0), myristic (C14:0), alpha-linolenic (C18:3ω-3), arachidonic (C20:4ω-6) and eicosapentaenoic (C20:5ω-3, EPA) acids, as well the sum of ω-3 PUFA, were significantly (*P<*0.05) lower in colostrum fat from caesarean than from vaginal deliveries (Table 6). In addition, oleic acid (C18:1ω-9) proportion, the sum of MUFA, the ratios of MUFA/SFA and of MUFA/PUFA, as well as the hypocholesterolaemic index (hI) value were significantly higher (*P* = 0.000, 0.001 0.015, 0.000 and 0.013, respectively) in colostrum fat from caesarean deliveries as compared to vaginal ones.

### Effect of customized centile and gender

To a further extent, the customized centile and gender effect on colostrum fat content and fatty acid profile was investigated (Table 7).

**Table 7:** Effect of customized centile on the individual FA proportions, sums and ratios as well as the LQI values of colostrum fat.

Groups		1	2	3			
	*P<*0.050	< 20	20–70	>70	'1–2'	'1–3'	'2–3'
Fat	0.00429	2.26±0.09	1.81±0.09	1.73±0.09	*	*	
C10:1	0.00740	0.09±0.01	0.10±0.01	0.10±0.01		*	*
C12:0	0.04546	5.80±0.28	4.70±0.21	5.01±0.17	*		
C13:0	0.02461	0.04±0.01	0.04±0.01	0.03±0.01	*		
*iso*C15:0	0.00353	0.03±0.00	0.01±0.00	0.02±0.00	*		*
C15:1ω-5	0.00849	0.06±0.01	0.08±0.01	0.05±0.00	*		*
*iso*C16:0	0.02388	0.44±0.03	0.34±0.04	0.32±0.03	*	*	
*iso*C17:0	0.03492	0.77±0.09	1.31±0.11	1.25±0.12	*	*	
C18:1*t*9	0.01239	0.01±0.01	0.01±0.00	0.03±0.01		*	*
C18:2*c*9*t*11	0.03994	0.07±0.00	0.17±0.03	0.12±0.02	*	*	*
C18:2*t*9*t*11	0.00474	0.04±0.01	0.07±0.02	0.03±0.00	*		*
C18:2*t*11*t*13	0.02049	0.06±0.01	0.09±0.01	0.06±0.01	*		*
C18:4ω-3	0.00204	0.11±0.00	0.13±0.01	0.10±0.00	*		*
C22:0	0.01118	0.05±0.01	0.08±0.01	0.06±0.01	*		
C23:0	0.03537	0.09±0.03	0.01±0.00	0.02±0.00	*	*	

Results represent mean values ± S.E.M. (standard error of mean)

Mean values in the same row with * differ significantly between two specific groups of customized centile (< 20, 20–70, >70) with *P<*0.05

The most interesting finding is that the colostrum fat content was found to inversely vary with the customized centile of the infants (Table 7). Furthermore, lauric, tridecanoic and *iso*-palmitic (*iso*C16:0) acids proportions were (*P<*0.05) highest in colostrum from mothers delivering infants at lower percentiles (<20). Moreover, the proportions of the *trans* fatty acids C18:1*t*11, C18:2*c*9*t*11, C18:2*t*9*t*11, and C18:2*t*11*t*13 showed significant variations among colostrum samples in terms of the infantile percentile.

The results of the present study suggested that the gender had no effect on the fatty acid profile of the colostrum fat, except for adrenic (C22:4ω-6) and pentadecenoic (C15:1ω-5) acids which were increased in the case of female new-borns.

## Conclusions

In conclusion, this study suggests that the fatty acid profile of colostrum fat was mainly affected by the nationality and age of the mother, rather than the mode of delivery and maternal BMI. Interestingly, the colostrum fat content was found to correlate with the customized centile of the infants in a reverse manner. The confirmation of the above results in greater cohorts may lead to specific dietary advices in breastfeeding women, in order to increase breast milk nutritional value.
